# The knowledge and reuse practices of researchers utilising government health information assets, Victoria, Australia, 2008–2020

**DOI:** 10.1371/journal.pone.0297396

**Published:** 2024-02-01

**Authors:** Merilyn Riley, Kerin Robinson, Monique F. Kilkenny, Sandra G. Leggat

**Affiliations:** 1 Department of Public Health, School of Psychology and Public Health, La Trobe University, Melbourne, Australia; 2 Stroke and Ageing Research, Department of Medicine, School of Clinical Sciences at Monash Health, Monash University, Victoria, Australia; 3 Stroke Division, The Florey Institute of Neuroscience and Mental Health, Melbourne Brain Centre, University of Melbourne, Victoria, Australia; 4 School of Public Health and Tropical Medicine, James Cook University, Townsville, Australia; Abo Akademi University: Abo Akademi, FINLAND

## Abstract

**Background:**

Using government health datasets for secondary purposes is widespread; however, little is known on researchers’ knowledge and reuse practices within Australia.

**Objectives:**

To explore researchers’ knowledge and experience of governance processes, and their data reuse practices, when using Victorian government health datasets for research between 2008–2020.

**Method:**

A cross-sectional quantitative survey was conducted with authors who utilised selected Victorian, Australia, government health datasets for peer-reviewed research published between 2008–2020. Information was collected on researchers’: data reuse practices; knowledge of government health information assets; perceptions of data trustworthiness for reuse; and demographic characteristics.

**Results:**

When researchers used government health datasets, 45% linked their data, 45% found the data access process easy and 27% found it difficult. Government-curated datasets were significantly more difficult to access compared to other-agency curated datasets (p = 0.009). Many respondents received their data in less than six months (58%), in aggregated or de-identified form (76%). Most reported performing their own data validation checks (70%). To assist in data reuse, almost 71% of researchers utilised (or created) contextual documentation, 69% a data dictionary, and 62% limitations documentation. Almost 20% of respondents were not aware if data quality information existed for the dataset they had accessed. Researchers reported data was managed by custodians with rigorous confidentiality/privacy processes (94%) and good data quality processes (76%), yet half lacked knowledge of what these processes entailed. Many respondents (78%) were unaware if dataset owners had obtained consent from the dataset subjects for research applications of the data.

**Conclusion:**

Confidentiality/privacy processes and quality control activities undertaken by data custodians were well-regarded. Many respondents included data linkage to additional government datasets in their research. Ease of data access was variable. Some documentation types were well provided and used, but improvement is required for the provision of data quality statements and limitations documentation. Provision of information on participants’ informed consent in a dataset is required.

## Introduction

The Association of Australian Medical Research Institutes (AAMRI) identified over 32,000 medical researchers undertaking research in Australia in 2019 [1]. Whilst many utilised primary data, there was an increasing focus on the reuse of health data [2, 3]. The extent of medical or health data reuse has not been quantified in Australia. Internationally, Neto et al. [4] identified an increase in the number of publications between 2010 to 2018 which cited the use of Open Government data sources, with health being the second most cited Open Government data domain.

Australia adopted the Open Data Charter principles formally in 2017 [5], committing to the reuse and sharing of non-sensitive government data as standard practice. In the state of Victoria, the collection, use, reuse and dissemination of human data for research purposes are governed under both national and state-based policies (e.g., the National Statement on Ethical Conduct in Human Research [6], national Privacy Principles [7], state Health Records Act [8], state Privacy and Data Protection Act [9]) and processes (e.g., passwords, encryption, secure portals) for protection and release of government health data. The Australian Government’s vision “to implement world class data and digital capabilities” [10, p.3] is slowly being implemented, but faces technological, cultural and resourcing challenges [11–13] that are also mirrored in the reuse of government data.

### Challenges of data reuse

Data reuse has many benefits [3, 4, 14], but also many challenges, particularly in health [15]. These include, but are not limited to: informed consent and de-identification of data; heterogenous data types; low data quality and inadequate standardisation; lack of technical infrastructure; and staff insufficiently qualified to facilitate data reuse [3, 16]. Additional challenges linked to researcher reuse include access and release of data, the researcher’s knowledge (or lack thereof) about data provenance, purposes, context, metadata, etc., and lack of available documentation to provide this information [17–19]. McGrath-Lone et al. [20] identified inconsistent understanding between organisations on the extent of data documentation and curation required for data to be considered research-ready. The data producer and their documentation, however, constitute only part of the data reuse scenario. Lee and Strong [21] identified three main players in data production and use: “the data collector, data custodian and data consumer” (p.17). Often, it is the data consumer, a researcher, who is responsible for the analysis, use and dissemination of information and knowledge produced from the data.

### Knowledge and infrastructure requirements for data reuse

Recognising the need for skilled researchers/staff to source, utilise, manage and analyse open-source data [22], the Australian Research Data Commons (ARDC) investigated the data discovery practices of researchers. Liu et al. [23] explored researchers’ approaches in sourcing, collecting and analysing data. This, in turn, helped inform data providers of information requirements researchers needed to make informed decisions about data reuse. Appropriate information must be available to answer questions of provenance, structure, definitions, quality, access and consent [24]. In parallel, researchers must have the *intent* to use, and sufficient skill to interrogate, manipulate and analyse these data and related documentation [25].

Access to government health data is no longer a simple process of an end-user requesting data access from the primary data producer or dataset custodian. This is reflective of Giddens’ [26] notion of time-space distanciation whereby data, in this case relating to patients treated, are extracted from the patient and, then, from their medical record and (re-)processed and re-cycled to become highly mobile and accessible data items that are remote from the patient-subject. Giddens found the associated “disembedding mechanisms” (p.20) to be dependent upon trust. Sexton et al., [27] in their Balance of Trust model, interpreted the access process as “abstract and faceless as standardised protocols for access take over from personal gatekeeping as a means of governing the tie between provider and researcher” (p.314). Given this “disembodied” process, it is important to understand researchers’ dataset knowledge and the key documentation they utilise, or do not utilise, to i) ensure the outputs from their research can be “trusted” i.e., are fit-for-purpose; and ii) determine if sufficient governance and infrastructure are available to support the meaningful reuse of government data.

In Australia, little research has been undertaken on investigating the experiences of researchers navigating governance and documentation processes for government health dataset reuse. Perrier et al. [28] investigated researchers’ data sharing and reuse practice perspectives and experiences in North America and Europe. Khan et al. [29] investigated data sharing and reuse practices across 20 broad Scopus disciplines. Hutchings et al. [30] completed a systematic literature review on how researchers and healthcare professionals view reuse of clinical trial and population-health administrative data. The findings from these studies [28–30] were generally supportive of data sharing and reuse, dependent upon the scientific discipline.

Whilst the international literature can provide an overview of the data reuse knowledge and practices overseas, this is not necessarily generalisable to the Australian context where researchers may face different challenges and barriers due to the local data landscape [31]. To advance the sharing and reuse of government health datasets in Australia, and to ensure that information assets are fit-for-purpose, it is important to understand the local knowledge and experiences of researchers in reusing government health data. This would enable governments and data custodians to identify and address any challenges or impediments for researchers for meaningful and accurate reuse of these data. The scope of this study focuses on government health information assets in the state of Victoria, Australia.

### Aim

It was the aim of this study to explore researchers’ knowledge and experience of governance processes, and their data reuse practices, when using Victorian government health datasets for research between 2008 and 2020.

## Method

### Study design

The study utilised a cross-sectional quantitative survey. The terms “datasets” and “information assets” have been used interchangeably in this paper.

### Sample

Riley et al. [32] identified 756 peer-review papers published between 2008–2020 which utilised selected Victorian Government Department of Health (The Department) information assets. These included 28 datasets containing person-level data related to health service provision. Corresponding author(s) of these publications formed the sample for the study.

### Inclusion and exclusion criteria

Where a corresponding author had written two or more papers using multiple within-scope datasets, the most recent study published within the study timeframe was the survey focus. If contact emails of first authors could not be validated then co-authors were approached for validation of the first author’s contact details or for a survey response. Contact details were verified via internet and social media search, where possible. If the contact emails could not be validated, authors were excluded from the study.

### Data collection

Authors were sent a survey participation email with attached Participant Information Consent Form and a link to an electronic Research Electronic Data Capture (REDCap) survey. There were two points of follow-up post initial contact, each approximately one month apart, from November 2022 to February 2023.

### Development of survey instrument

The survey was designed in four parts: (A) Researcher’s data reuse practice; (B) Knowledge of government health information assets; (C) Perceptions of data trustworthiness for reuse; and (D) Demographic characteristics. The survey instrument contained 49 items requiring close-ended responses; four of these contained branching logic to capture extended free text responses (S1 File).

Sections A and B of the survey were informed by literature on barriers and facilitators to data reuse (S1 Table). Section C was informed by the works of Wang and Strong [33], Caro et al. [34], Wilkinson et al. [35] and Yoon and Lee [36]. Some of the items seeking demographic data in section D were based upon questions utilised by Kim and Yoon [18].

Questions related to researchers’ use-practice focussed on data access, data provision, data linkage, data validation and the following data documentation (i.e., information-categories): contextual; data dictionary (meta-data); data quality statement; and limitation(s) document (S1 File).

Participants responded to statements on findability and usefulness of documentation on a 5-point Likert scale, their level of agreement ranging from ‘*very easy to locate’* to ‘*very difficult to locate’* and *‘very useful’* to *‘not useful at all’*.

A pilot survey was conducted with ten researchers experienced in the use of government datasets, but whose publications were outside the scope of the current survey. Feedback was provided on clarity, appropriateness, and question content. Pilot survey responses were not incorporated in the main data collection.

This paper focuses on Parts A and B of the survey. Parts C and D of the survey, including demographic characteristics, are included in another paper (pending publication).

### Analysis

Descriptive statistics were completed using IBM SPSS Version 28. Respondents could nominate to complete information on up to two datasets. Missing responses or “Did not Use” responses were excluded from the denominator for the questions on ease of documentation findability and documentation usefulness measured by a Likert scale. Chi-square, α = 0.05, was calculated in OpenEpi Version 3.01, to investigate associations for categorical variables. Not stated or missing responses were excluded from the chi-square calculations. Fisher’s exact test was utilised when cell numbers were less than five [37].

For the analyses of ease of access, each dataset was broadly categorised into:

1. population-health–“factors that influence the health of population groups or whole populations” [38]; or
2. administrative–“routine management of service provision” [39].

Health datasets were also categorised by “government-curated” datasets and “other-agency” curated datasets. Other-agencies included registries, research agencies, screening services, and professional associations.

The qualitative open-ended survey responses underwent a three-phase analysis. Thematic analysis using an inductive approach was initially undertaken [40]. Relevant components of multi-part responses containing different foci were separated and included in the analysis; therefore, the frequency of comments exceeded the number of respondents. Comments were manually reviewed and assigned to associated themes in an Excel spreadsheet. Once assigned to themes, manual sentiment analysis [41, 42] was undertaken, with comments separately categorised by orientation, as positive (use of affirmative adjectives/descriptor), neutral (statement of fact) or negative (unfavourable adjective/descriptor). The comments were then classified according to data custodian (owner), i.e., either government- or other agency- curated. A second reviewer independently reviewed all three categorisations.

### Ethics

Ethics approval was provided by the La Trobe University Human Research Ethics Committee [HEC21401].

## Results

There were 62/399 respondents to the survey after exclusion of two responses with datasets outside scope (15.5%) (S1 Fig). Fifty respondents completed all questions in the survey (full response) and 12 respondents attempted Part A and/or Part B only (partial response) (S2 Table). Twelve respondents completed the survey on behalf of two datasets, providing a potential denominator of 74 datasets for some questions.

### Information obtained from contact list (not survey)

REDCap uses anonymisation which enables researchers to identify participants who have responded to the survey—either fully or partially–but does not allow linkage of a survey response to a specific respondent. The contact list in REDCap showed that 64 respondents “attempted” the survey. The REDCap contact database provided details of the year of publication and respondent employment organisation.

Over 60% of responses (*n* = 39/62) related to papers published between 2017–2020, 24% (*n* = 15/62) to studies published between 2013–2016 and 14% (*n* = 8/62) to studies published between 2008–2012. Most respondents were employed by universities (50%, *n* = 31/62) or hospitals (40%, *n* = 25/62), with the remainder employed in government, registry/screening agencies or research institutes.

### What do researchers do?

#### Publications between 2008–2020

**Fig 1:**
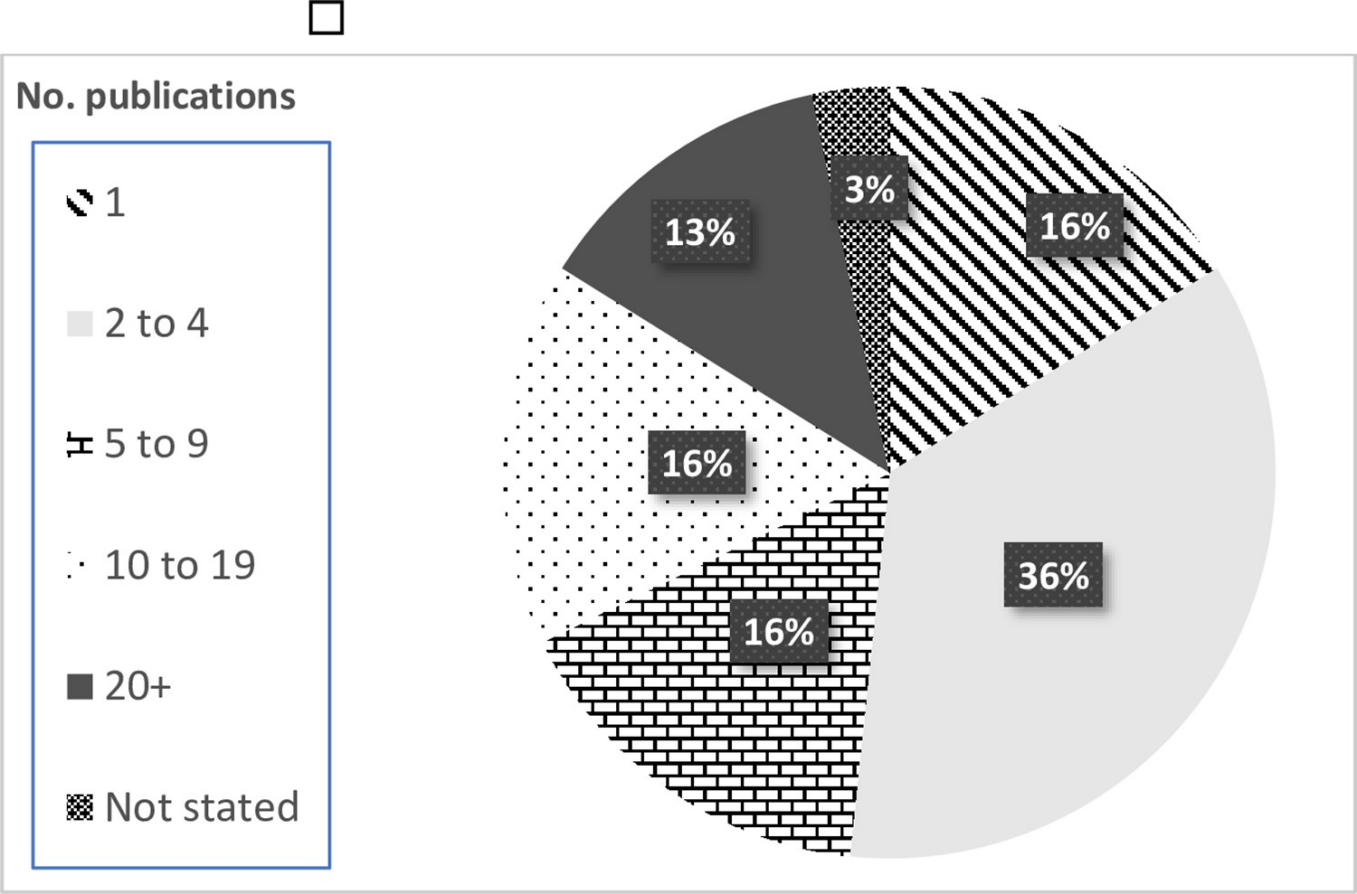
Number of publications completed by respondents using government health datasets between 2008–2020.

Respondents were asked the number of research studies they had published between 2008–2020 that utilised *any* government health datasets (not just within-scope datasets) (Fig 1). One in three participants (29%, *n* = 18/62) reported having completed 10 or more publications during this period.

#### Frequency of utilisation of within-scope datasets for survey responses

Completed surveys related to 18/28 within-scope datasets (S1 Appendix). The three most frequently used datasets were: Victorian Admitted Episode Dataset (VAED) (26%, *n* = 19/74); Australian and New Zealand Intensive Care Society Adult Patient Database (ANZICS APD) (12%, *n* = 9/74); and Victorian Emergency Minimum Dataset (VEMD) (12%, *n* = 9/74). Just under half (45%, *n* = 28/62) of respondents received their within-scope dataset-1 already linked to other, mostly government, population-health or administrative datasets (Table 1).

**Table 1:** Additional health data sources linked to within-scope dataset-1, 2008–2020.

Linked data source	Number^#^
National Death Index (NDI)	9
Medicare Benefits Scheme (MBS)	4
Pharmaceutical Benefits Scheme (PBS)	3
Population Level Analysis and Reporting (POLAR)	2
Ambulance Services	2
Australasian Rehabilitation Outcomes Centre (AROC)	2
National Aged Care Data Clearinghouse (NACDAC)	0
Department of Veteran’s Affairs (DVA)	0
Other*	21
**Total**	**43**

* Australian Bureau of Statistics Census, Registries (e.g., joint replacement), Coroners, Bureau of Meteorology, population health studies (e.g., Longitudinal study of Australian children), National Disability Insurance Scheme, Victorian Electoral roll, Registry of Birth, Deaths and Marriages, prenatal diagnosis and IVF clinical data. #This question was not asked for dataset-2.

#### Request for data access

Almost 45% (*n* = 33/74) of respondents identified the experience of requesting data access as easy/very easy, whilst 27% (*n =* 20/74) indicated the process was difficult/very difficult. There was no statistically significant difference in ease of requesting data access between health datasets categorised as administrative or population-health (Table 2). There was, however, a statistically significant difference in the perceived difficulty requesting access for government-curated datasets compared to other-agency curated datasets (χ^2^ = 6.78, p = 0.009).

**Table 2:** Ease of requesting data access by dataset-category and custodian-category for dataset-1 and dataset-2 combined.

	Easy/Very Easy		Neutral		Difficult/Very difficult		Not stated*		Total	^χ2^ P-value
***Dataset-category***	**No.**	***%***	**No.**	***%***	**No.**	**%**	**No.**	**%**	** **	**0.37**
Administrative	12	*37*.*5*	5	*15*.*6*	11	*34*.*4*	*4*	*12*.*5*	32	
Population-health	21	*51*.*2*	9	*22*.*0*	9	*22*.*0*	*2*	*4*.*9*	41	
Unknown	0	*0*	0	*0*	0	*0*	*1*	*100*	1	
***Custodian-category***		* *		* *		* *	* *	* *		**0.009**
Government-curated	15	*32*.*6*	10	*21*.*7*	16	*34*.*8*	*5*	*10*.*9*	46	
Other-agency-curated	18	*66*.*7*	4	*14*.*8*	4	*14*.*8*	*1*	*3*.*7*	27	
Unknown	0	*0*	0	*0*	0	*0*	*1*	*100*	1	

*Excluded from calculation of chi-square values

**Fig 2:**
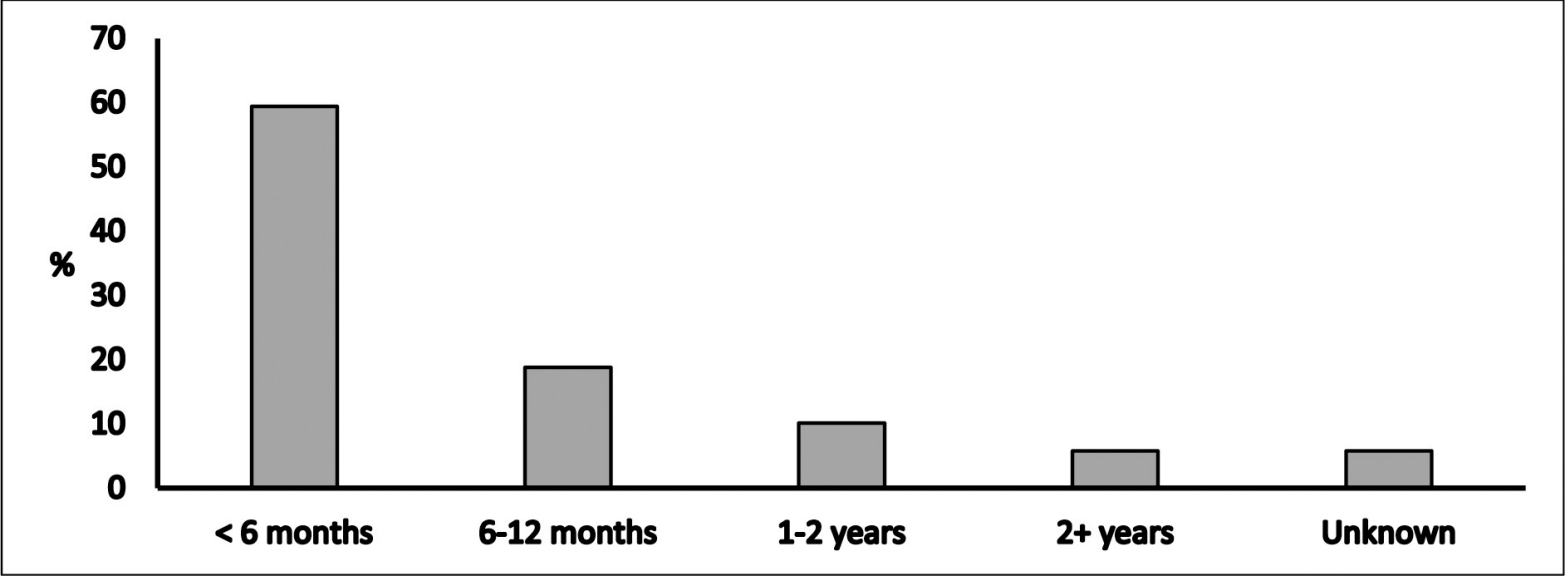
Time to receipt of data from request lodgement.

For two of the most frequently used government-curated datasets, almost half of the respondents identified requesting data access as easy/very easy (i.e., *n* = 7/18 and *n* = 3/7, respectively) whilst the other half identified it as difficult/very difficult, confirming the variable nature of ease of access. There was one “other-agency” curated dataset where 78% (*n* = 7/9) of respondents consistently identified the access process as easy to follow. Most respondents received their requested data in less than six months from lodging their request (Fig 2).

#### Privacy/Confidentiality and security

Most respondents (76%, *n* = 56/74) reported they received either aggregated or de-identified individual records without possibility of participant re-identification (S3 Table). Data providers used various security methods when sending data to the respondents (S4 Table). Only two respondents (3%) indicated no security methods had been used. Almost 45% (*n* = 33/74) of all security methods used by data providers to send data to researchers involved password protection, often along with other methods (e.g., encryption, secure portal, etc.) (S4 Table).

#### Data validation processes

Most respondents (almost 70%, *n* = 51/74) performed their own data validation checks, with almost 18% (*n* = 13/74) performing no additional checks and 14% (*n* = 10/74) providing no response to this question (S5 Table). Eleven percent (*n* = 8/74) undertook only one validation activity (i.e., checking missing values or checking duplicates or recoding to new variables or other-not-specified). Many respondents (58%, *n* = 43/74) used a *combination* of validation checks including checking missing values, duplicates and inconsistencies, recoding variables, and validating calculations. Additional validation activities included: a validation study; checked 10% against original medical record; rule-based ordering; user-written aggregation; mix of checking dates and confirming diagnostic information.

#### Use of selected information asset documentation (i.e., information-categories)

**Fig 3:**
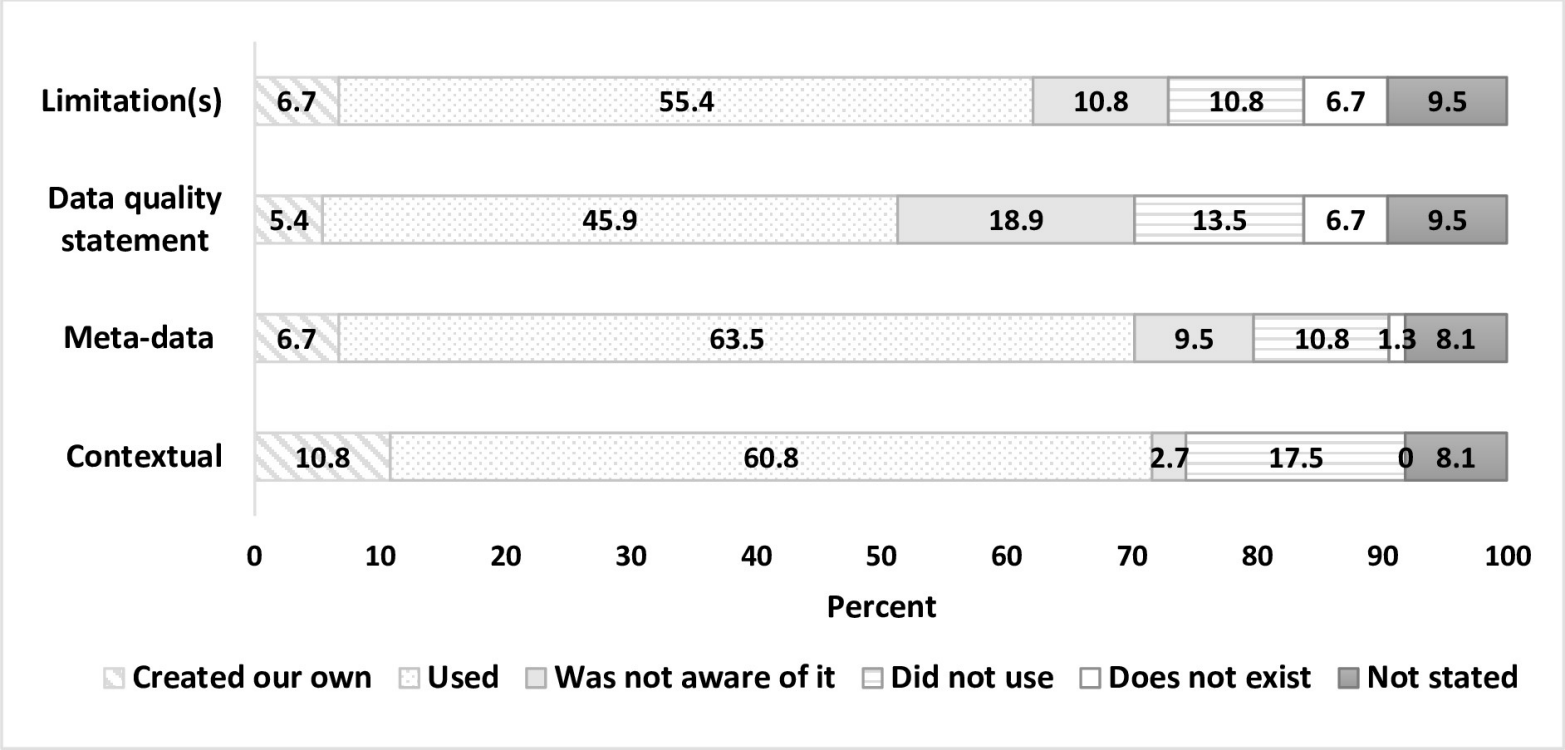
Awareness and/or use of specified information-categories for each dataset (*n* = 74).

Respondents were asked questions about their knowledge and use-practices in relation to selected documentation (i.e., information-categories) to assist with data reuse for each dataset: contextual documentation; data dictionary (meta-data); data quality statement(s); and limitation(s) document (Fig 3).

More than 50% of all respondents utilised, or created their own, contextual documentation, a meta-data dictionary, data quality statement and limitations document (Fig 3). For each information-category, at least 20% of respondents did not use the information-categories, were not aware of their existence or stated they did not exist. Almost 20% of respondents were not aware if data quality information existed for the dataset they had accessed.

**Fig 4:**
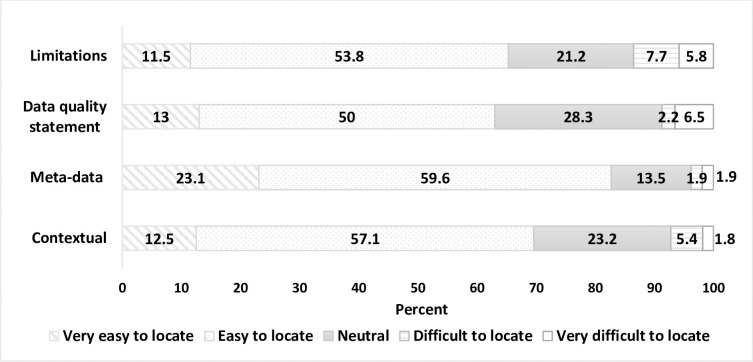
Findability of specified information-categories.

More than 50% of respondents who used each information-category indicated they were easy/very easy to locate (contextual—70% (*n* = 39/56), meta-data dictionary—83% (*n* = 41/52), data quality statement—63% (*n* = 29/46), limitations document—65% (*n =* 34/52)). Limitations documentation had the highest proportion of the four categories reported as difficult/very difficult to locate (13.5%, *n*- = 7/52) (Fig 4).

**Fig 5:**
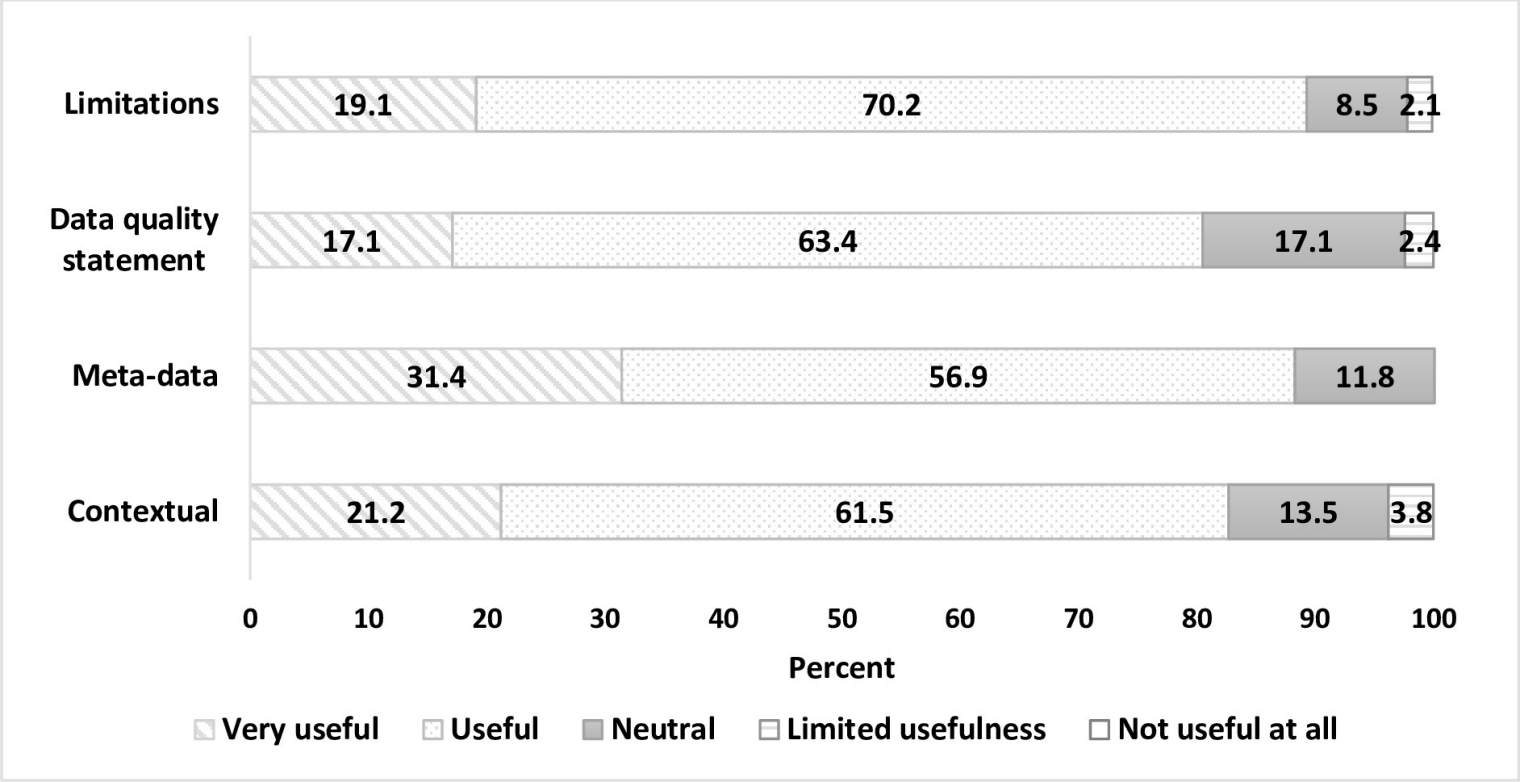
Usefulness of specified information-categories.

More than 80% of respondents identified all information-categories as useful/very useful (contextual– 83% (*n* = 43/52), meta-data dictionary– 88% (*n* = 45/51), data quality statement– 80% (*n* = 33/41), and limitations document– 89% (*n* = 42/47)) (Fig 5).

### What do researchers know?

Respondents were asked to report their knowledge about aspects of information governance surrounding datasets they utilised (Table 3). Only 63 respondents for dataset-1 and dataset-2 combined responded to these questions. Overall, respondents had very good knowledge of the data custodian’s identity (either organisational or an individual) and the nature of the dataset (i.e., voluntary or mandatory reporting). Forty-three percent of respondents (*n* = 27/63) had associations with the data custodian either through present or past employment, previous research or professional affiliation.

**Table 3:** Researchers’ reported knowledge of selected information governance processes about government-health datasets utilised, 2008–2020, dataset-1 and dataset-2 combined.

What researchers said they know*	What researchers said they don’t know*
• Identity of data custodian (98%, *n* = 62/63)	• If dataset participant provided informed consent (78%, *n* = 49/63)
• Mandatory or voluntary nature of dataset (82.5%, *n* = 52/63)	
• Perception of rigorous data quality processes (76%, *n* = 48/63)	• Details of data curation/quality processes (51%, *n* = 32/63)
• Perception of rigorous confidentiality/privacy and security processes (94%, *n* = 59/63)	• Security measures utilised to protect data sent to researchers from providers (21%, *n* = 14/69)

*Proportion of overall respondents who answered the question; 11 responses were missing

Respondents perceived most datasets (94%) to have appropriate governance processes to ensure data confidentiality/privacy and security. Over three-quarters of respondents (78%) did not know, or were unsure, whether the subjects whose data were included in the government dataset(s) had provided informed consent for inclusion of their data.

#### Quality process(es) for datasets for “fitness for purpose”

Three-quarters of respondents (*n* = 48/63) perceived data quality processes surrounding their dataset(s) to be sufficiently rigorous to provide ‘fit-for-purpose’ data. Only one in two (*n* = 32/63) reported being confident in explaining the details of the data quality/curation processes.

Forty-nine respondents provided free-text reasons for their response to the question *“Are data quality processes sufficiently rigorous to provide a ‘fit-for-purpose’ dataset*?*”* Seven major themes emerged: data validation and quality processes; custodial staff attributes; coverage; reputation and output; knowledge; purpose; and content (S6 Table). The predominant theme was ‘data validation and quality processes’ (Table 4).

**Table 4:** Examples from the analysis of qualitative responses to the question *“Are data quality processes sufficiently rigorous to provide a ‘fit-for-purpose’ dataset*?*”*.

Examples of qualitative responses*	Theme (% of all comments)	Orientation	Data Custodian/ Owner
• *‘Meticulous process[es] to ensure data are obtained and entered by the Registry’* [Participant 15]	Data validation and quality processes (42%)	Positive	Other-agency
• ‘*There are a large number of checks undertaken on the data before it is released’* [Participant 40]		Neutral	Government
• *‘Not the processes conducted by the Department of Health or the VAED—I don’t believe there are any’* [Participant 17]		Negative	Government
• *‘It has been used extensively in prior research*, *leading to impactful outputs and outcomes’* [Participant 35]	Reputation and output (13%)	Positive	Other-agency
• *‘Errors are likely to be random and unlikely to be a source of bias”* [Participant 31]		Neutral	Other-agency
*‘Some variables are known to be less accurate’* [Participant 33]		Negative	Government
• *‘The data are easy to use once you understand what they mean’* [Participant 23].	Knowledge (12%)	Positive	Government
‘*Our ICU contributes data to this dataset & we implement similar quality checks to those of other ICUs*’ [Participant 18]		Neutral	Other-agency
• *‘The data is relevant to our work and reflects real world’ [*Participant 24]	Content (10%)	Positive	Other-agency
• *‘Fields relevant for these reasons [funding]*, *such as timing of events*, *are quite reliable*. *Other data*, *such as diagnoses*, *are unreliable as specific diagnoses (such as ‘lobar pneumonia’)*, *but more reliable in larger aggregated groups (such as respiratory diseases)’* [Participant 7]		Neutral	Government
*The survey items were not fit-for-purpose for our research*, *and the psychometric properties of the items had not been verified’* [Participant 16]		Negative	Government
‘*The data was used for costing/billing for hospitals*. *There are problems*, *but it gave an idea of trends’* [Participant 26]	Purpose (9%)	Neutral	Government
• *‘Staff involved [being] experienced*, *rigorous in their approach’* [Participant 3]	Custodial staff attributes (7%)	Positive	Government
*‘For the VAED there are trained coders entering the information from patient notes’* [Participant 10]		Neutral	Government
*‘Generally*, *the data provided was what I needed*, *although more participant and hospital details would have added to the value (e*.*g*., *age was in 5 year groups*, *which in young children is less than ideal)’* [Participant 11]	Coverage (6%)	Neutral	Government

Overall, 19 qualitative comments were classified as positive, 45 as neutral and four as negative. There was no difference in the proportion of positive comments when government-curated datasets were compared with other-agency curated datasets (26% versus 24%, respectively). There were more negative comments related to government-curated datasets than to other-agency curated datasets (11.5% versus 0%, respectively).

The majority of the qualitative responses to ***“Are data quality processes sufficiently rigorous to provide a ‘fit-for-purpose’ dataset*?*”*** were categorised as neutral rather than positive, despite 75% of respondents providing an affirmative answer to this question. However, given the high proportion of respondents who considered the data quality processes to be rigorous, in most cases the “neutral” statements (e.g., “audited annually” [Participant 2]), were interpreted as positive because these actions were reported by respondents as reasons for regarding datasets as ‘fit-for-purpose’.

## Discussion

This study explored researchers’ knowledge of government health datasets which they utilised for secondary research purposes and their specific reuse-practices related to these data. Whilst the response rate was small, the results provide clear insight into the experience and knowledge of experienced academic and clinical researchers in Victoria, Australia, from 2008–2020, which has not previously been explored.

### Year(s) of publications

Year of publication of within-scope datasets ranged from 2008 to 2020. An “infodemic” comprising proliferation of big data, data warehousing, increased data linkage [43], and the Open Data Era [44], has seen many changes in data management practices during this period [45]. In the earlier years of the study timeframe, data were more likely to be locally managed within separate units with The Department (personal knowledge of the authors). Subsequent years saw organisational re-structures within The Department [46]; the expansion of the Centre for Victorian Data Linkage (CVDL) [47], a centralised entity to manage release of health information and trusted data linkage; and the establishment of the Victorian Agency for Health Information (VAHI) [48]. The CVDL joined VAHI in 2021 [49]. This reflects Sexton et al’s. [27] findings of a transition from a personal relationship-based data access model to a more impersonal centralised service across the whole-of-health with researchers’ liaising with VAHI staff responsible for data retrieval, and not with the data producers, custodians or data experts in the field.

### Data access

Ease of accessibility is a motivator in researchers’ satisfaction with data reuse practices [50]. It is also a factor that can discourage the reuse of data [51]. Historically, access to Australian government data has been problematic due to “lack of trust by both data custodians and users” [52, p.2] Andrew et al. [53] described the challenges in obtaining cross-jurisdictional data in Australia for data-linkage purposes, with the overall process taking more than two years. In the United Kingdom, Williamson et al. [54] described the process of accessing routine healthcare data, which involved 15 applications and/or agreements and took over three years.

Riley et al’s. [55] documentary analysis of the availability of website information-categories involving the within-scope datasets identified that almost 70% of dataset websites contained information on the access process. Our survey demonstrated, however, that less than half of the respondents reported the process for requesting data access to be easy. Given the documentary analysis findings [55], we would expect a higher proportion of survey respondents to find the access process easier if it was only related to documentation availability. In our survey we did not ask reasons why the process for requesting access was difficult; however, other researchers have reported ease of access can be related to factors such as data type (e.g., identifying versus aggregated), the access portal (e.g., openness), external factors (e.g., legal/legislative compliance issues), public engagement (e.g., acceptability of data release) [56], or resourcing issues (e.g., cost of infrastructure to sustain sharing and reuse of government data) [57].

Whilst ease of access was not significantly more difficult for the less experienced, compared to the more experienced researchers in our study, it was significantly more difficult for government-curated datasets compared to other-agency curated datasets. This supports our finding that one of the “other-agency” curated datasets was consistently identified as ‘easy to access’ compared to the other datasets. Level of government documentation may impact upon the ease with which instructions on accessing government datasets may be followed. Bureaucratic organisations such as hospitals, legal firms, governments, etc. “often have reputations for communicating poorly” [58, p.336]. The use of “plain language and word choice” [59], as outlined in the Australian Government style manual, can make complex processes easy to follow.

**Recommendation 1**: Government data custodians should audit their website documentation on data access to ensure information is presented in clear plain language, and that it reflects current government access processes.

### Data linkage

Data linkage is common in contemporary research practice and plays a major role in utilisation of government health datasets [60]. Since 2009, with the establishment of Australia’s national Population Health Research Network (PHRN), data linkage facilities have become progressively embedded within government entities. The number of peer-reviewed publications utilising data linkage undertaken by the PHRN-funded data linkage unit more than tripled between 2009–10 and 2016–17 [42]. Tew et al. [61] demonstrated the significant increase in the use of linked hospital data for secondary purposes subsequent to when Western Australia (from mid-late 1990s) and New South Wales (from mid-late 2000s) introduced data linkage units. Currently in Victoria, the CVDL routinely links 25 Victorian health and human services datasets: the Integrated Data Resource [IDR]) is available through a centralised request hub as a de-identified resource for research purposes [62]. This routine data linkage was not readily available during all of our study period (2008–2020). In our study, the importance of health data linkage practices for governments was demonstrated by almost half of the subject-researchers who indicated they had linked data for their research, mostly to government administrative datasets.

### Data privacy, confidentiality and security

Almost all respondents perceived the privacy/confidentiality and security processes for the datasets they utilised to be rigorous. This was substantiated by Riley et al’s. [55] documentary analysis which revealed that 17/25 datasets provided information about privacy/confidentiality and security issues for data release. Most survey respondents were satisfied with existing policies and governing legislation [6–9] and processes (e.g., passwords, encryption, secure portals) for protection and release of government health data. This may have been influenced by the high proportion of experienced researchers in the study who are more likely to support reuse/sharing of government health data than younger, less experienced researchers [15].

Despite this confidence in existing privacy/confidentiality and security processes, many respondents lacked knowledge on whether participant informed consent was obtained for the datasets they reused. Riley et al. [55] identified that only 7/25 websites clearly identified whether participant consent was required for inclusion of data within the dataset. Reasons for this lack of knowledge on participant informed consent were not collected in this survey. Hutchings et al’s. systematic review [15] demonstrated the diverse opinions on whether or not there is a need for additional informed consent when data are utilised for reuse purposes, and various studies have proposed a range of different mechanisms to manage this [63, 64]. To fill this knowledge gap, information on informed consent should be included on dataset websites.

**Recommendation 2:** A plain language statement relating to the requirement (or not) for participant informed consent should be clearly available on all dataset websites.

### Knowledge and use of information asset data quality controls and validation techniques

Liu et al. [23] reported that the importance of data quality attributes related largely to methodological rigour, institutional reputation, and ability to trace data origins. This was consistent with our survey outcomes, specifically that all respondents in our study were able to identify the data custodian of the information asset they utilised, and three-quarters affirmed that custodian-initiated data quality processes were rigorous. Our finding that only half of the respondents could explain the data quality processes related to their datasets may reflect the depth of researcher experience with the datasets or the relationships between the respondent and the data custodians. Overwhelmingly, the respondents’ explanations for perceived dataset “fitness-for-purpose” were linked to data validation and quality processes undertaken by the custodian, even if respondents did not know what they were. It is important for researchers to become familiar with “routine data production activities” [21, p.15] as it provides them with knowledge to interrogate and solve potential data quality problems.

**Recommendation 3:** A data flow diagram of the curation and quality control processes should accompany each government dataset for ease of user reference.

### Use and knowledge of dataset information-categories

Whilst this study did not explore researchers’ knowledge prior to accessing a dataset, it did explore their knowledge about the existence, findability and usefulness of specific types of information-categories which may have contributed to their data reuse decision, and practice [65].

**(i) Contextual information**. Most respondents were aware of the context of their dataset(s). All indicated awareness of contextual documentation, although a small number created their own, presumably derived from other available information. This was consistent with the findings of Riley et al. [55], that almost all datasets provided contextual information on their websites. It is encouraging that most researchers were aware of the context of the government health datasets they reused, and that this information was well-documented on dataset websites.

It is possible, however, for website documentation to be available and still not be useful. As part of their data utility model, Gordon et al. [66] proposed a graded framework moving from bronze (the lowest level) through to platinum (the highest level). In their system, a dataset would be measured as bronze if, for example, the dataset source was available, and it would be graded as platinum if there was opportunity to “view earlier versions… review the impact of each stage of data cleaning” (p.7). Given the usefulness of contextual information, the attendant documentation needs to be reviewed regularly to ensure currency. Quality of the documentation is as important as its availability.

**Recommendation 4**: Data custodians should regularly review the contextual information provided on their websites to ensure its accessibility, currency, and usefulness.

**(ii) Data dictionary (meta-data)**. Reliable meta-data has been identified as an important motivator in promoting data reuse [67]. “High-quality metadata that support understanding and reuse and cross domains are a critical antidote to information entropy, particularly as it supports reuse of the data” [68, p.1]. Riley et al. [55] found that almost 60% of health datasets included a meta-data dictionary on their website; however, 10% of the current survey respondents were unaware of its existence and 10% did not use it. The survey identified many respondents who acknowledged the importance of meta-data in the promotion of data use; notwithstanding this, there is room for improvement to reach a higher level of interoperability.
**(iii) Data quality statement or information**. The importance of understanding data quality within the context of data reuse has been previously identified [14, 18]; however, our findings show that only half of the respondents utilised data quality information. Similarly, Riley et al. [55] identified a large proportion (83%) of health datasets that did not provide data quality information on their websites. This information-category had the highest proportion from the four information-categories of respondents who were not aware if data quality information existed for their dataset. This finding is not unique to this study. For example, Canaway et al. [69] identified similar findings in their study on primary care datasets, where 30% of data custodians were unaware if any data quality assessments/activities had been applied to their data.

Respondents may rely upon a knowledge source other than website documentation to provide data quality information. Notably, informal peer networks often provide a pathway to either unpublished information or access to ‘inside information’ from peers who have previously used the dataset [14]. Forty percent of respondents indicated an association with the data custodian either through current or past employment or previous research, which may also have provided them with “insider” knowledge of the dataset data quality.

**Recommendation 5**: Data custodians should provide access to routine data quality information either through data quality templates/statements or links to published data quality reports or peer-reviewed publications.

**(iv) Data limitation documentation**. A data reuser does not usually know the data as intimately as the data producer/collector [70], nor is the associated documentation always sufficiently detailed to provide the necessary information. Liu et al. [23] identified that access to data producers gave data reusers support in engaging with the dataset and “making sense of it” (p.3). The lack of information outlining limitations of datasets for reuse identified in a documentary analysis [55], and the one-third of our survey respondents who did not use such documentation, highlighted gaps in both the provision and the use of limitations documentation for data reuse.

**Recommendation 6**: Plan language statements of the potential limitations in use of datasets for purposes other than those for which they were originally collected should be readily accessible.

### Limitations

Response bias may be present in our study due to the low response rate; however, the respondents were broadly representative of clinical and other heath researchers in Victoria. Restriction of the survey to researchers using Victorian datasets *only* may have affected the generalisability of the findings; however, the diverse organisations represented by the respondents minimised the potential lack of external validity. Recall bias may have been present because of the time lag, potentially up to 14 years in some instances, between the respondents’ conduct of the relevant research and their completion of the survey. To minimise this bias, focus was placed on the latest within-scope publication authored by each respondent; hence, 60% of responses related to publications between 2017–2020 rather than the earlier years of the study period. Changes in data management processes over time (i.e., between 2008–2020) may have confounded results, although this was minimised by the inclusion of the respondents’ most recent within-scope publication. We were unable to analyse by time because the date of publication was not included as a field in the survey; however, a proxy date from the respondent contact list was utilised.

The items for Parts A and B of the survey were based upon literature that addressed barriers and facilitators of data reuse, and other trust studies. The survey did not contain items to cover all issues but was representative of the issues raised in these papers. A reliability assessment of the survey was not conducted due to the small sample size.

Categorisation of free-text comments using sentiment analysis presented challenges by the very nature of their “subjectivity” [41]; however, studies have demonstrated that manual sentiment analysis is more reliable than either automated or dictionary-based approaches [42].

## Conclusion

This study explored researchers’ knowledge and use-practices of governance processes and specific documentation information-categories surrounding Victorian government health information assets. It quantitatively demonstrated that: governance processes for maintaining privacy/confidentiality and quality control activities undertaken by data custodians are well-regarded; researchers link their data with government datasets; ease of requesting data access is variable; some documentation types are reasonably well provided and used; improvement is required for the provision and use of data quality statements and limitation documentation; and provision of information on dataset subjects’ informed consent is required. Six recommendations have been provided to inform the research-readiness for reuse of government health datasets. Uptake of these recommendations by government and data custodians should enhance both the knowledge and experience of researchers when utilising government health information assets for reuse purposes.

## Supporting information

S1 File Health data use and trustworthiness (Parts A and B). (DOCX)

S1 Fig Survey response pathway. (DOCX)

S1 Table Potential survey items for Parts A and B sourced from literature on barriers and facilitators for data reuse. (DOCX)

S2 Table Responses to survey parts. (DOCX)

S3 Table Method to restrict identification of participants, dataset-1 and dataset-2 combined. (DOCX)

S4 Table Security methods utilised by data providers to send data to researchers, dataset-1 and dataset-2 combined. (DOCX)

S5 Table Data validation activities undertaken by researchers, dataset-1 and dataset-2 combined. (DOCX)

S6 Table Qualitative responses to question: “Are data quality processes sufficiently rigorous to provide a ‘fit-for-purpose’ dataset?”. (DOCX)

S1 Appendix Datasets for which a survey was completed (dataset-1 and dataset-2 combined). (DOCX)

## References

[1] Health research in Australia—AAMRI [Internet]. AAMRI. 2023 [cited 2023 Jun 4]. Available from: https://www.aamri.org.au/health-medical-research/fast-facts-on-medical-research.

[2] Australian Institute of Health and Welfare [AIHW]. Australia’s health 2018: A secondary use of health information [cited 2023 Jun 4]. Available from: https://www.google.com/search?client=firefox-bd&q=Australia%27s+health+2018%3A+Secondary+use+of+health+information.

[3] Huston P, Edge V, Bernier E. Reaping the benefits of Open Data in public health. *Canada Communicable Disease Report*. 2019 Oct 3;45(10):252–6. doi: 10.14745/ccdr.v45i10a01 ; PMCID: PMC6781855. 31647060 PMC6781855

[4] Neto AJA, Neves DF, Santos LC, Junior MCR, do Nascimento RPC. Open Government data usage overview. *Proceedings of the Euro American Conference on Telematics and Information Systems*. 2018 Nov 12; Article No. 29:1–8. doi: 10.1145/3293614.3293619

[5] Open data charter. Government adopters [Internet]. n.d. [cited 2023 July 19]. Available from: https://opendatacharter.net/government-adopters/.

[6] National Health and Medical Research Council. National Statement on Ethical Conduct in Human Research (2007)—Updated 2018. NHMRC [Internet]. NHMRC.gov.au. 2018. [cited 2023 July 11]. Available from: https://www.nhmrc.gov.au/about-us/publications/national-statement-ethical-conduct-human-research-2007-updated-2018.

[7] Office of the Australian Information Commissioner [OAIC]. Australian Privacy Principles [Internet]. Office of the Australian Information Commissioner. 2023. [cited 2023 July 14]. Available from: https://www.oaic.gov.au/privacy/australian-privacy-principles.

[8] Health Records Act 2001 [Internet]. www.legislation.vic.gov.au. 2022. [cited 2023 July 15]. Available from: https://www.legislation.vic.gov.au/in-force/acts/health-records-act-2001/047.

[9] Privacy and Data Protection Act 2014 [Internet]. Vic.gov.au. 2014. [cited 2023 July 15]. Available from: https://www.legislation.vic.gov.au/in-force/acts/privacy-and-data-protection-act-2014/028.

[10] Australian Government. Data and digital Government strategy. The data and digital vision for a world-leading (Australian Public Service) APS to 2030. [internet]. 2021 [cited 2023 July 20]. Available from: https://www.dataanddigital.gov.au/.

[11] Janssen M, Charalabidis Yand Zuiderwijk A. Benefits, adoption barriers and myths of open data and open government. *Information Systems Management*, 29. 2012: 4: 258–268. doi: 10.1080/10580530.2012.716740

[12] Ubaldi B. Open government data. OECD working papers on public governance [Internet]. 2013 May 27;22:8–10. [cited 2023 July 20]. Available from: https://www.oecd-ilibrary.org/governance/open-government-data_5k46bj4f03s7-en.

[13] Henninger M. Reforms to counter a culture of secrecy: Open government in Australia. *Government Information Quarterly*. 2018;35:398–407. 10.1016/j.giq.2018.03.003.

[14] Sielemann K, Hafner A, Pucker B. The reuse of public datasets in the life sciences: potential risks and rewards. *PeerJ*. 2020 Sep 22;8:e9954. doi: 10.7717/peerj.9954 ; PMCID: PMC7518187. 33024631 PMC7518187

[15] Hutchings E, Loomes M, Butow P, Boyle FM. A systematic literature review of attitudes towards secondary use and sharing of health administrative and clinical trial data: a focus on consent. *Systematic Reviews*. 2021 Dec; 10:1–44. 10.1186/s13643-021-01663-z. 33941282 PMC8094598

[16] Hulsen T, Jamuar SS, Moody AR, Karnes JH, Varga O, Hedensted S, et al. From Big Data to Precision Medicine. *Frontiers in Medicine* [Internet]. 2019 Mar 1;6. Available from: https://www.ncbi.nlm.nih.gov/pmc/articles/PMC6405506/. doi: 10.3389/fmed.2019.00034 30881956 PMC6405506

[17] Curty RG, Crowston K, Specht A, Grant BW, Dalton ED. Attitudes and norms affecting scientists’ data reuse. Sugimoto CR, editor. *PLOS ONE*. 2017 Dec 27;12(12):e0189288. doi: 10.1371/journal.pone.0189288 29281658 PMC5744933

[18] Kim Y, Yoon A. Scientists’ data reuse behaviors: A multilevel analysis. *Journal of the Association for Information Science and Technology*. 2017 Sep 12;68(12):2709–19. doi: 10.1108/OIR-09-2020-0431

[19] Imker HJ, Luong H, Mischo WH, Schlembach MC, Wiley C. An examination of data reuse practices within highly cited articles of faculty at a research university. *Journal of Academic Librarianship*. 2021 Jul 1;47(4):102369–9. doi: 10.1016/j.acalib.2021.102369

[20] Mc Grath-Lone LM, Jay MA, Blackburn R, Gordon E, Zylbersztejn A, Wiljaars L et al. What makes administrative data "research-ready"? A systematic review and thematic analysis of published literature. *International Journal of Population Data Science* 2022 Apr;7(1):1718. doi: 10.23889/ijpds.v6i1.1718 ; PMCID: PMC9052961. 35520099 PMC9052961

[21] Lee YW, Strong DM. Knowing-why about data processes and data quality. *Journal of Management Information Systems*. 2003;20(3):13–39. doi: 10.1080/07421222.2003.11045775

[22] Johnson M, Jain R, Brennan-Tonetta P, Swartz E, Silver D, Paolini J, et al. Impact of Big Data and Artificial Intelligence on Industry: Developing a workforce roadmap for a data driven economy. *Global Journal of Flexible Systems Management*. 2021 May 25;22(3):197–217. doi: 10.1007/s40171-021-00272-y

[23] Liu YH, Wu M, Power M, Burton A. Elicitation of data discovery contexts: An interview study. 2022 Oct 10; doi: 10.5281/zenodo.7179526

[24] Gilbert R, Lafferty R, Hagger-Johnson G, Harron K, Zhang LC, Smith P, et al. GUILD: GUidance for Information about Linking Data sets†. *Journal of Public Health*. 2017 Mar 28;40(1):191–8. doi: 10.1093/pubmed/fdx037 ; PMCID: PMC5896589. 28369581 PMC5896589

[25] LaFlamme M, Poetz M, Spichtinger D. Seeing oneself as a data reuser: How subjectification activates the drivers of data reuse in science. *PLOS One*. 2022 Aug 18;17(8):e0272153–3. doi: 10.1371/journal.pone.0272153 35980953 PMC9387815

[26] Giddens A. *Modernity and self-identity*: *self and society in the late modern age*. Cambridge: Polity Press; 1991.

[27] Sexton A, Shepherd E, Duke-Williams O, Eveleigh A. A balance of trust in the use of government administrative data. *Archival Science*. 2017 Oct 10;17(4):305–30. doi: 10.1007/s10502-017-9281-4

[28] Perrier L, Blondal E, MacDonald H. The views, perspectives, and experiences of academic researchers with data sharing and reuse: A meta-synthesis. Dorta-González P, editor. *PLOS ONE*. 2020 Feb 27;15(2):e0229182. doi: 10.1371/journal.pone.0229182 32106224 PMC7046208

[29] Khan N, Thelwall M, Kousha K. Data sharing and reuse practices: disciplinary differences and improvements needed. *Online Information Review*. 2023 Feb 7. doi: 10.1108/oir-08-2021-0423

[30] Hutchings E, Loomes M, Butow P, Boyle FM. A systematic literature review of researchers’ and healthcare professionals’ attitudes towards the secondary use and sharing of health administrative and clinical trial data. *Systematic Reviews*. 2020 Oct 12;9(1). doi: 10.1186/s13643-020-01485-5 33046097 PMC7552458

[31] Hardy K, Maurushat A. Opening up government data for Big Data analysis and public benefit. *Computer Law & Security Review*. 2017;33(1): 30–37.

[32] Riley M, Robinson K, Kilkenny MF, Leggat SG. The suitability of government health information assets for secondary use in research: A fit-for-purpose analysis. *Health Information Management Journal*. 2022 Apr 26;183335832210783. Epub ahead of print. doi: 10.1177/18333583221078377 . 35471919

[33] Wang RY, Strong DM. Beyond Accuracy: What data quality means to data consumers. *Journal of Management Information Systems*. 1996 Mar;12(4):5–33. doi: 10.1080/07421222.1996.11518099

[34] Caro A, Calero C, Caballero I, Piattini M. A proposal for a set of attributes relevant for Web portal data quality. *Software Quality Journal*. 2008 Mar 15;16(4):513–42. doi: 10.1007/s11219-008-9046-7

[35] Wilkinson MD, Dumontier M, Aalbersberg IjJ, Appleton G, Axton M, Baak A, et al. The FAIR Guiding Principles for scientific data management and stewardship. *Scientific Data*. 2016 Mar 15;3(1). doi: 10.1038/sdata.2016.18 26978244 PMC4792175

[36] Yoon A, Lee YY. Factors of trust in data reuse. *Online Information Review*. 2019 Nov 11;43(7):1245–62. doi: 10.1108/OIR-01-2019-0014

[37] Kim HY. Statistical notes for clinical researchers: Chi-squared test and Fisher’s exact test. *Restorative Dentistry and Endodontics*. 2017;42(2): 152–155. doi: 10.5395/rde.2017.42.2.152 28503482 PMC5426219

[38] NSW Government. What is population health research?—Population health research and evaluation [Internet]. www.health.nsw.gov.au. [cited 2023, August 14]. Available from: https://www.health.nsw.gov.au/research/Pages/what-is-population-health-research.aspx.

[39] Cadarette SM and Wong L. An introduction to health care administrative data. *The Canadian Journal of Hospital Pharmacy*. 2015; 68(3): 232–237. doi: 10.4212/cjhp.v68i3.1457 26157185 PMC4485511

[40] Braun V and Clarke V. Using thematic analysis in psychology. *Qualitative Research in Psychology*. 2008: 3(2):77–101. doi: 10.1191/1478088706qp063oa

[41] Feldman R. Techniques and applications for sentiment analysis. *Communications of the ACM*. 2013 Apr 1;56(4):82–89. https://dl.acm.org/doi/fullHtml/10.1145/2436256.2436274?casa_token=oztdy-yVbkEAAAAA:DvUGCAhCE5cOH8Olj-hW6Xqd_5zxI7PjZDGitucTl_EAI-SFDS7PwrSSEcBy3RCKod37JCGyxw5s.

[42] van Atteveldt W, van der Velden MACG and Boukes M. The validity of sentiment analysis: Comparing manual annotation, crowd-coding, dictionary approaches, and machine learning algorithms. *Communication Methods and Measures* 2021;(15)2:121–140. doi: 10.1080/19312458.2020.1869198

[43] Young A, Flack F. Recent trends in the use of linked data in Australia. *Australian Health Review*. 2018;42(5):584. doi: 10.1071/AH18014 30145995

[44] Department of Finance. Australian Data Strategy [Internet]. Finance.gov.au. 2022. Available from: https://www.finance.gov.au/publications/strategy/australian-data-strategy (cited 2023 24 April).

[45] Krahe MA, Toohey J, Wolski M, Scuffham PA, Reilly S. Research data management in practice: Results from a cross-sectional survey of health and medical researchers from an academic institution in Australia. *Health Information Management Journal*. 2019 Mar 11;49(2–3):108–16. Epub 2019 Mar 11. doi: 10.1177/1833358319831318 . 30857424

[46] Sakkal P and Dow A. “Too big”: Government to separate Health and Human Services departments [Internet]. *The Age*. 2020 [cited 2023 Jun 5]. Available from: https://www.theage.com.au/politics/victoria/government-to-separate-health-and-human-services-departments-20201130-p56j3w.html.

[47] Victorian Agency for Health Information. The Centre for Victorian Data Linkage [Internet]. Vic.gov.au. 2021. [cited 2023 April 10]. Available from: https://www.health.vic.gov.au/reporting-planning-data/the-centre-for-victorian-data-linkage.

[48] Safer Care Victoria. Health Services Data and Centre for Victorian Data Linkage join VAHI. [Internet]. Vic.gov.au 2021. [cited 2023 August 21]. Available from https://www.safercare.vic.gov.au/news-and-media/health-services-data-and-centre-for-victorian-data-linkage-join-vahi.

[49] Victorian Agency for Health Information [VAHI]. About the Centre for Victorian Data Linkage. [Internet] n.d. [cited 2023 July 14]. Available from: https://vahi.vic.gov.au/ourwork/data-linkage/about.).

[50] Faniel IM, Kriesberg A, Yakel E. Social scientists’ satisfaction with data reuse. *Journal of the Association for Information Science and Technology*. 2015 May 4;67(6):1404–16. doi: 10.1002/asi.23480

[51] York J. Seeking Equilibrium in data reuse: A study of knowledge satisficing [Internet]. deepblue.lib.umich.edu. 2022 [cited 21 Aug 2023]. Available from: https://deepblue.lib.umich.edu/handle/2027.42/174439.

[52] Productivity Commission. Data availability and use, inquiry report. Canberra, Productivity Commission, 2017. [cited 2023 August 21]. https://www.pc.gov.au/inquiries/completed/data-access/report.

[53] Andrew NE, Sundararajan V, Thrift AG et al. Addressing the challenges of cross-jurisdictional data linkage between a national clinical quality registry and government-held health data. *Aust N Z J Public Health*. 2016 Oct;40(5):436–442. Epub 2016 Sep 13. doi: 10.1111/1753-6405.12576 . 27625174

[54] Williamson K, Nimegeer A & Lean M. Navigating data governance approvals to use routine health and social care data to evidence the hidden population with severe obesity: A case study from a clinical academic’s perspective. *Journal of Research in Nursing* 2022;27(7): 623–636. doi: 10.1177/17449871221122040 36405806 PMC9669932

[55] Riley M, Kilkenny MF, Robinson K, Leggat SG. An audit of government population-health information assets’ websites to identify ’research-readiness’ documentation, Victoria, Australia. *Health Information Management Journal* 2023;0(0) doi: 10.1177/18333583231197756 PMC11705755 37702287

[56] Gorsky M, Mold A. Documentary analysis. Ch.7 in Pope C and Mays N (Eds) *Qualitative research in health care*, 4^th^ edn. Hoboken, NJ: Wiley Blackwell; 2020;83–96.

[57] Organisation for Economic Co-operation and Development (OECD). Risks and challenges of data access and sharing. [Internet] 2019. [cited 2023 July 27]. Available from: https://www.oecd-ilibrary.org/sites/15c62f9c-en/index.html?itemId=/content/component/15c62f9c-en.

[58] Matveeva N, Moosally M and Willerton R. Plain language in the Twenty-first century: Introduction to the special issue on plain language. *IEEE Transactions on Professional Communication*. 2017 Dec.:60(4):336–442. doi: 10.1109/TPC.2017.2759619

[59] Australian Government. Style Manual. Plain language and word choice. [Internet]. 2023. [cited 2023 July 19]. Available at https://www.stylemanual.gov.au/writing-and-designing-content/clear-language-and-writing-style/plain-language-and-word-choice.

[60] Smith M, Flack F. Data linkage in Australia: The first 50 years. *International Journal of Environmental Research and Public Health*. 2021 Oct 28;18(21):11339. doi: 10.3390/ijerph182111339 34769852 PMC8583508

[61] Tew M, Dalziel KM, Petrie DJ et al. Growth of linked hospital data use in Australia: a systematic review. *Australian Health Review* 2016;41(4): 394–400. 10.1071/AH16034. 27444270

[62] Victorian State Government Department of Health. Datasets available in the Centre for Victorian Data Linkage’s Integrated Data Resource. [Internet] 2021, April. [cited 2023 July 23]. Available from: https://www.health.vic.gov.au › files › factsheets.

[63] Ploug T, Holm S. Meta consent: a flexible and autonomous way of obtaining informed consent for secondary research *BMJ* 2015; 350: h2146 doi: 10.1136/bmj.h2146 25952952

[64] Gefenas E., Lekstutiene J., Lukaseviciene V. et al. Controversies between regulations of research ethics and protection of personal data: informed consent at a cross-road. *Med Health Care and Philos* 25, 23–30 (2022). 10.1007/s11019-021-10060-1. 34787769 PMC8595272

[65] Wang X, Duan Q, Liang M. Understanding the process of data reuse: An extensive review. *Journal for the Association for Information Science and Technology*. 2021 Sep 1;72(9):1161–82. doi: 10.1002/asi.24483

[66] Gordon B, Barrett J, Fennessy C, Cake C, Milward A, Irwin C, et al. Development of a data utility framework to support effective health data curation. *BMJ Health & Care Informatics*. 2021 May;28(1):e100303. doi: 10.1136/bmjhci-2020-100303 33980500 PMC8117992

[67] Kim Y. A study of the roles of metadata standard and repository in science, technology, engineering and mathematics researchers’ data reuse. *Online Information Review*. 2022; 45(7):1306–1321. doi: 10.1108/OIR-09-2020-0431

[68] Habermann T. Metadata and reuse: Antidotes to information entropy. *Patterns*. 2020 Apr;1(1):100004. doi: 10.1016/j.patter.2020.100004 33205081 PMC7660388

[69] Canaway R, Boyle D, Manski-Nankervis JA et al. Identifying primary care datasets and perspectives on their secondary use: a survey of Australian data users and custodians. *BMC Med Inform Decis Mak*. 2022 Apr 6;22(1):94. doi: 10.1186/s12911-022-01830-9 35387634 PMC8988328

[70] Pasquetto IV, Borgman CL, Wofford MF. Uses and reuses of scientific data: The data creators’ advantage. *Harvard Data Science Review* 12. 2019 Nov 15;1(2). 10.1162/99608f92.fc14bf2d.

