# Supplementary figures and images for: The knowledge and reuse practices of researchers utilising government health information assets, Victoria, Australia, 2008–2020
Source: PLoS One. 2024 Feb 1;19(2):e0297396. doi: 10.1371/journal.pone.0297396 (PMC10833579; doi:10.1371/journal.pone.0297396)

SUPPLEMENTARY MATERIAL

S1 Survey. Health Data Use and Trustworthiness (Parts A and B)


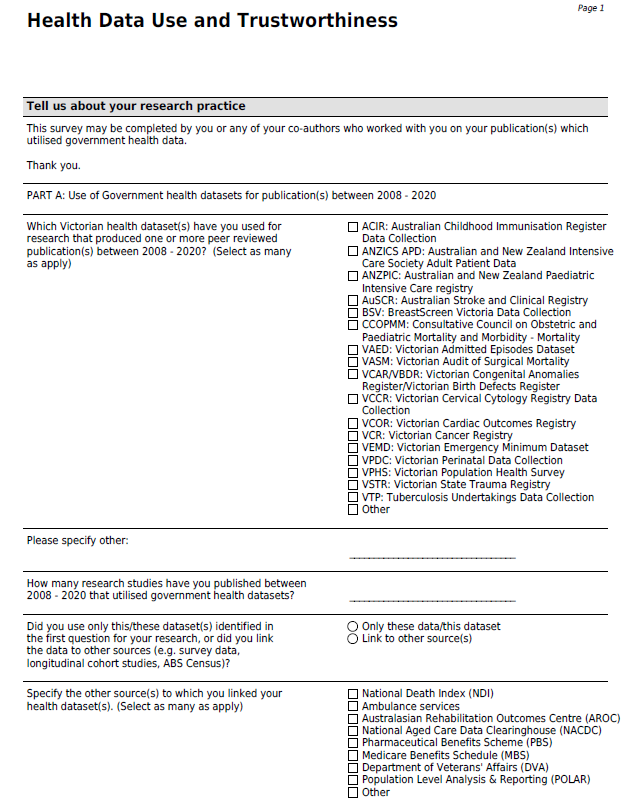


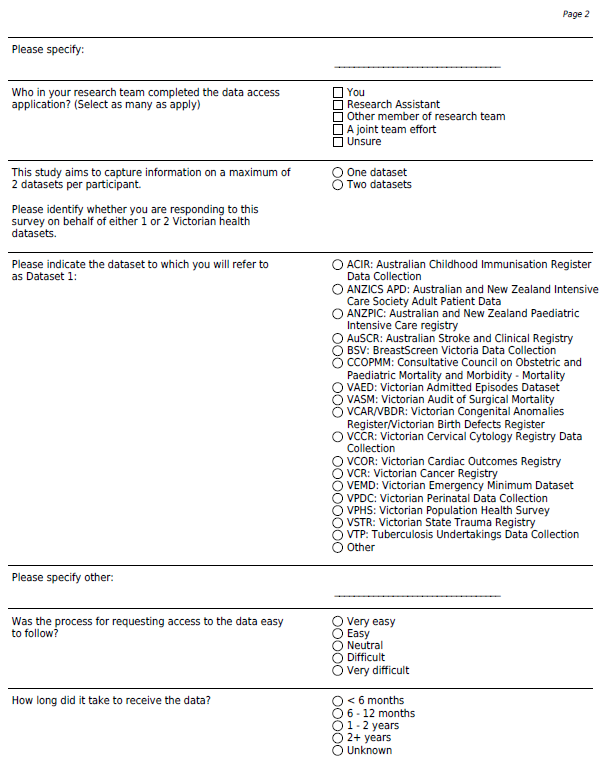


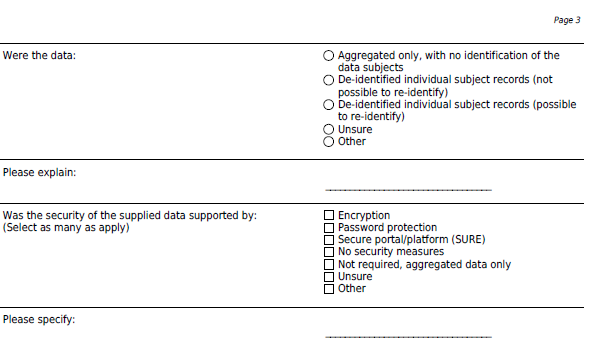


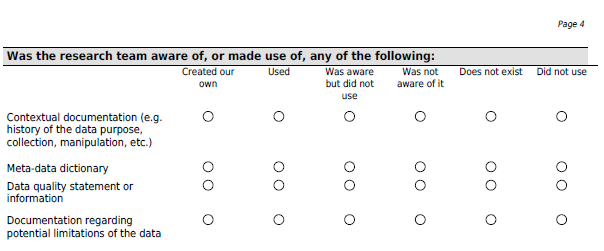


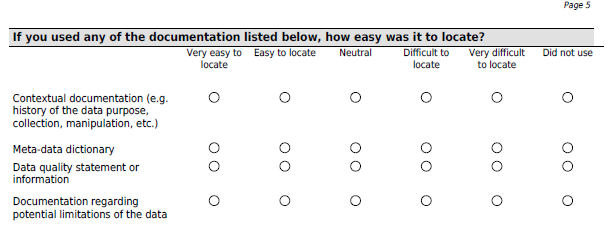


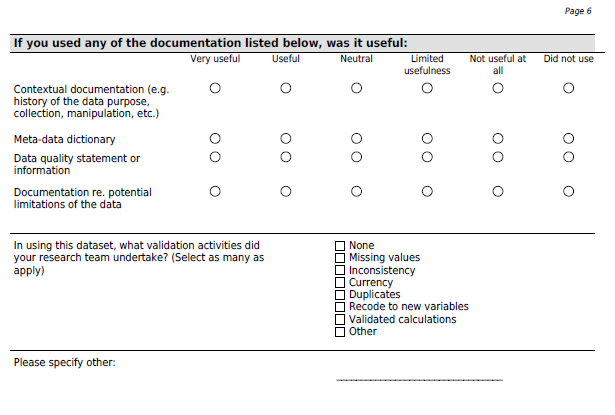


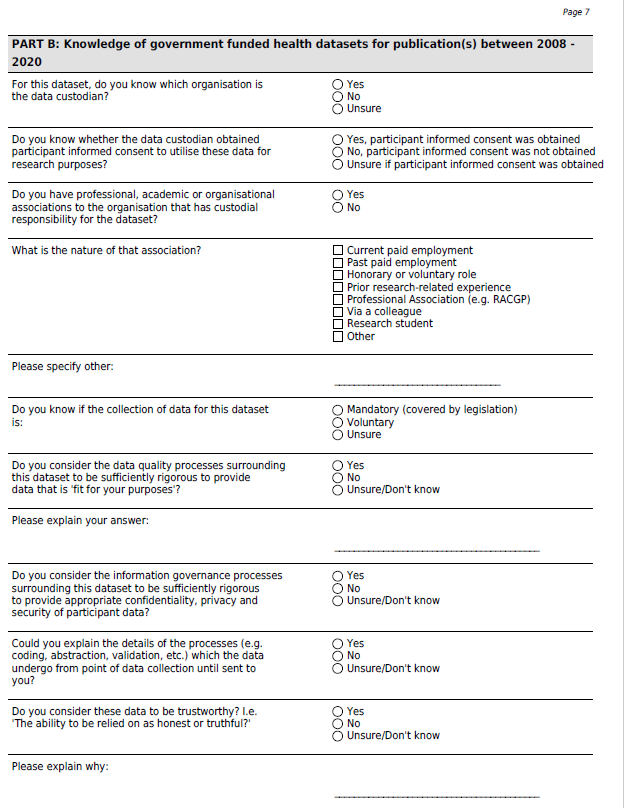


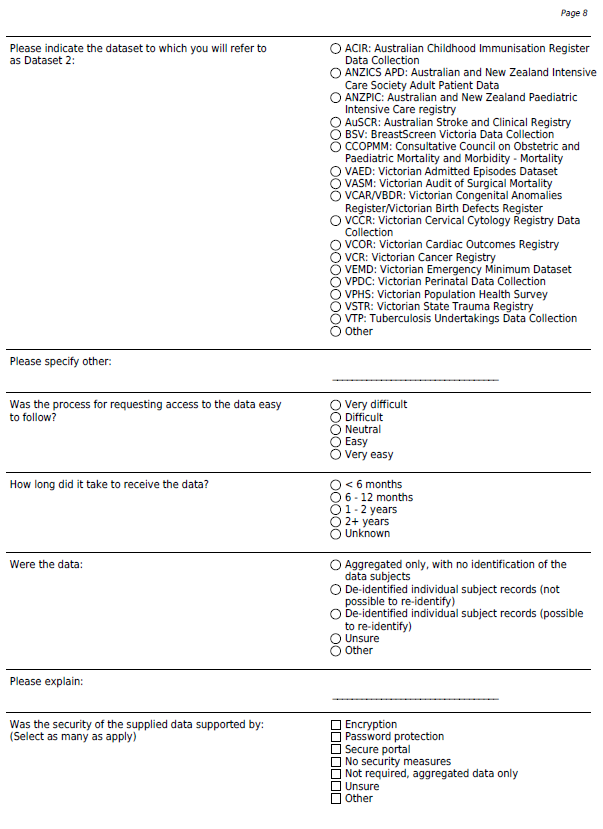


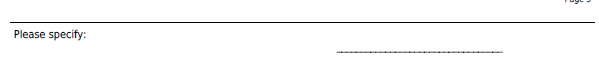


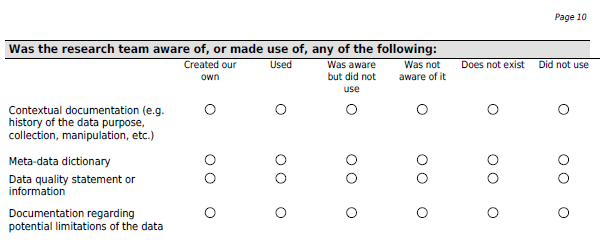


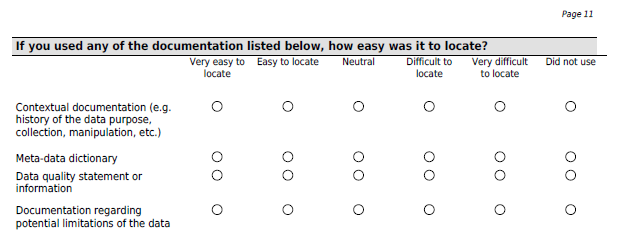


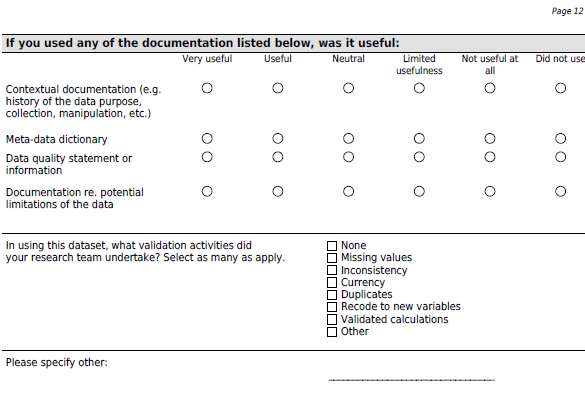


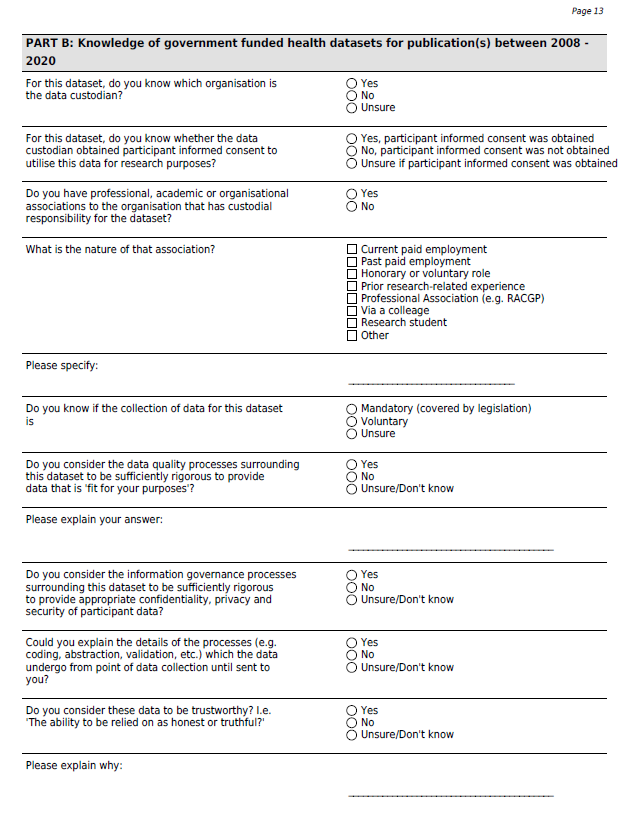

Supplement: S1 File — (DOCX) [file pone.0297396.s001.docx]
