# Supplementary material for: The knowledge and reuse practices of researchers utilising government health information assets, Victoria, Australia, 2008–2020
Source: PLoS One. 2024 Feb 1;19(2):e0297396. doi: 10.1371/journal.pone.0297396 (PMC10833579; doi:10.1371/journal.pone.0297396)
Supplement: S1 Fig — (DOCX) [file pone.0297396.s002.docx]

SUPPLEMENTARY MATERIAL


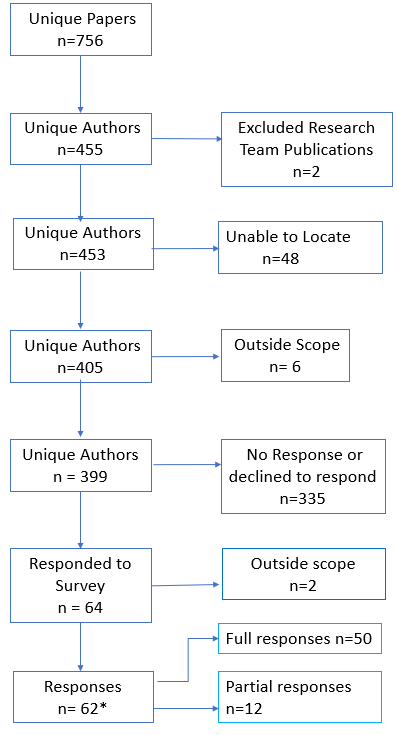


****50 respondents completed information on 1 dataset and 12 respondents completed information on 2 datasets -> 74 responses for some questions.***

**S1 Figure. Survey response pathway**
