# Supplementary material for: The knowledge and reuse practices of researchers utilising government health information assets, Victoria, Australia, 2008–2020
Source: PLoS One. 2024 Feb 1;19(2):e0297396. doi: 10.1371/journal.pone.0297396 (PMC10833579; doi:10.1371/journal.pone.0297396)
Supplement: S1 Table — (DOCX) [file pone.0297396.s003.docx]

SUPPLEMENTARY MATERIAL

**S1 Table: Potential survey items for Parts A and B sourced from literature on barriers and facilitators for data reuse**

| **Possible survey constructs** | **Data reuse facilitators and barriers** | **Other trust papers** |
| --- | --- | --- |
| **USER** |  |  |
| ***Perceived effort*** | Kim and Yoon^1^ |  |
| ***Pre-disposition to trust*** |  | Bauer & Freitag^2^ |
| * personality |  | Mayer et al.^3^; Kelton et al.^4^ |
| *socio-demographic issues | Tenopir et al.^5^ |  |
| ***Experience*** |  |  |
| *inexperience | Kim and Yoon1; Perrier et al.^6^ |  |
| ***Motivation/effort required*** | Sexton et al.^7^ ; Perrier et al.^6^ |  |
|  |  |  |
| **COMMUNITY/WORK CULTURE** |  |  |
| ***Organisational support re reuse (technical)*** | Imker et al.^8^; Kim & Yoon^1^ |  |
| ***Work/Discipline culture*** |  |  |
| *attitudes to data reuse | Curty et al.^9^; Sielemann et al.^10^; Imker et al.^8^; Perrier et al.^6^ |  |
| *some discipline share more than others | Tenopir et al.^5^; Perrier et al.^6^ |  |
| ***Mentorship/collaboration available/stakeholder engagement*** | Curty et al.^9^; Kim and Yoon^1^ |  |
|  |  |  |
| **DATA (INTRNSIC AND EXTRINSIC)** |  |  |
| ***Fit for purpose/relevant - reason for collection*** | Curty et al.^9^ | Kelton et al.^4^ |
| ***Data quality (accuracy, complete, coherent)*** | Curty et al.^9^; Imker et al.^8^; Sielemann et al.^10^; Perrier et al.^6^ |  |
| ***Format*** | Curty et al.^9^; Imker et al.^8^; |  |
| ***Source (objectivity)*** |  |  |
| *Less value than original data/proximity to original source; data collected as byproduct of clinical care -decreased quality/what is worth sharing? | Kim & Yoon^1^ Sexton et al.^7^;  Safran et al.^11^; Perrier et al.^6^ |  |
| ***Widely used (peer-reviewed)*** | Sielemann et al.^10^ |  |
|  |  |  |
| **DATA MANAGEMENT ISSUES** |  |  |
| ***Documentation/Guidelines for standardised use*** | Imker et al.^8^; Sielemann et al.^10^ |  |
| *contextual information | Faniel et al.^12^; Perrier et al.^6^ |  |
| *meta-data | Curty et al.^9^; Perrier et al.^6^ |  |
| *lack of data quality information | Perrier et al.^6^ |  |
| ***Locating data*** | Curty et al.^9^ |  |
| ***Ease of access*** | Imker et al.^8^; Kim & Yoon^7^; Sielemann et al.^10^; |  |
| ***Security*** | Cumyn et al.^13^; |  |
| ***Participant consent*** | Cumyn et al.^13^ |  |
| ***Cost & efficiency*** | Curty et al.^9^; Kim & Yoon^7^; Sielemann et al.^10^; |  |
| ***Copyright infringement/intellectual property*** | Kim and Yoon^7^; Perrier et al.^6^ |  |
|  |  |  |
| **DATA PROVIDER** |  |  |
| ***Nature of organisation/reputation*** |  | Kelton et al.^4^ |
| ***Privacy/confidentiality*** | Curty et al.^9^; Sielemann et al.^10^; Perrier et al.^6^ |  |
| ***Transparency in process*** | Safran^13^; Sielemann et al.^10^ |  |
| ***Commitment of organisation to high quality and appropriate data management practices*** | Sielemann et al.^10^ |  |
| ***Relationship or experience with organisation*** | Sexton et al.^7^ |  |
| ***Collaboration issues*** | Curty et al.^9^ |  |
