# Supplementary material for: The knowledge and reuse practices of researchers utilising government health information assets, Victoria, Australia, 2008–2020
Source: PLoS One. 2024 Feb 1;19(2):e0297396. doi: 10.1371/journal.pone.0297396 (PMC10833579; doi:10.1371/journal.pone.0297396)
Supplement: S2 Table — (DOCX) [file pone.0297396.s004.docx]

SUPPLEMENTARY MATERIAL

S2 Table. Responses to survey parts

|  | **Respondents** | |  |
| --- | --- | --- | --- |
|  | **Dataset 1** | **Dataset 2** | **Total Denominator** |
| Part A: Use of government-health datasets for publications between 2008-2020 | 62 | 12 | 74 |
| Part B: Knowledge of government-funded health datasets for publication(s) between 2008-2020 | 62 | 12 | 74 |
| Part C: What influences YOUR perception of data and information trustworthiness for reuse purposes | 51 | | 51 |
| Part D: Demographic characteristics of researcher | 50 | | 50 |
