# Supplementary material for: The knowledge and reuse practices of researchers utilising government health information assets, Victoria, Australia, 2008–2020
Source: PLoS One. 2024 Feb 1;19(2):e0297396. doi: 10.1371/journal.pone.0297396 (PMC10833579; doi:10.1371/journal.pone.0297396)
Supplement: S3 Table — (DOCX) [file pone.0297396.s005.docx]

SUPPLEMENTARY MATERIAL

**S3 Table. Method to restrict identification of participants, dataset-1 and dataset-2 combined**

|  | **Number** | **Percent** |
| --- | --- | --- |
| ***Data format*** |  | |
| Aggregated only | 9 | *12.2* |
| De-identified individual records | 47 | *63.5* |
| De-identified individual records- re-identify | 8 | *10.8* |
| Unsure | 2 | *2.7* |
| Other* | 3 | *4.1* |
| Not stated | 5 | *6.7* |
| **Total** | **74** | ***100.0*** |

***’Other’ format was not specified by respondents.*
