# Supplementary material for: The knowledge and reuse practices of researchers utilising government health information assets, Victoria, Australia, 2008–2020
Source: PLoS One. 2024 Feb 1;19(2):e0297396. doi: 10.1371/journal.pone.0297396 (PMC10833579; doi:10.1371/journal.pone.0297396)
Supplement: S4 Table — (DOCX) [file pone.0297396.s006.docx]

SUPPLEMENTARY MATERIAL

**S4 Table. Security methods utilised by data providers to send data to researchers, dataset-1 and dataset-2 combined**

|  | **Number** | **Percent** |
| --- | --- | --- |
| **Method of security** |  | |
| Encryption only | 2 | *2.7* |
| Password protection only | 15 | *20.3* |
| Secure portal/SURE only | 10 | *13.5* |
| Encryption & password | 6 | *8.1* |
| Password & secure portal | 4 | *5.4* |
| Encryption, password & SURE | 6 | *8.1* |
| Encryption, password, SURE & other | 1 | *1.4* |
| Encryption & SURE portal | 1 | *1.4* |
| Password, other | 1 | *1.4* |
| No security measures | 2 | *2.7* |
| Not required - aggregate data only | 4 | *5.4* |
| Other only* | 3 | *4.0* |
| Unsure | 14 | *18.9* |
| Not stated | 5 | *6.7* |
| **Total** | **74** | ***100*** |

*#’Other’ format was not specified by respondents.*
