# Supplementary material for: The knowledge and reuse practices of researchers utilising government health information assets, Victoria, Australia, 2008–2020
Source: PLoS One. 2024 Feb 1;19(2):e0297396. doi: 10.1371/journal.pone.0297396 (PMC10833579; doi:10.1371/journal.pone.0297396)
Supplement: S5 Table — (DOCX) [file pone.0297396.s007.docx]

SUPPLEMENTARY MATERIAL

**S5 Table. Data validation activities undertaken by researcher, dataset 1 and dataset 2 combined**

|  | **Frequency** | ***Percent*** |
| --- | --- | --- |
| None | 13 | *17.6* |
| Missing values only | 6 | *8.1* |
| Duplicates only | 0 | *0.0* |
| Recode to new variables only | 1 | *1.4* |
| Other activity-not listed | 1 | *1.4* |
| ***The most frequent combinations are displayed below:*** | | |
| 1. Missing values, inconsistencies, duplicates, recode, validate calculations | 9 | *12.2* |
| 1. Missing values, inconsistencies, duplicates, recode | 5 | *6.8* |
| 1. Missing values, duplicates | 4 | *5.4* |
| 1. All listed activities | 3 | *4.0* |
| Other combinations of listed activities | 22 | *29.7* |
| Not stated | 10 | *13.5* |
| **Total** | **74** | ***100.0*** |
