# Supplementary material for: The knowledge and reuse practices of researchers utilising government health information assets, Victoria, Australia, 2008–2020
Source: PLoS One. 2024 Feb 1;19(2):e0297396. doi: 10.1371/journal.pone.0297396 (PMC10833579; doi:10.1371/journal.pone.0297396)
Supplement: S6 Table — (DOCX) [file pone.0297396.s008.docx]

**Table 6: Qualitative responses to question: “Are data quality processes sufficiently rigorous to provide a ‘fit-for-purpose’ dataset?”**

| Processes | Staffing | Coverage | Reputation & Outputs | Knowledge | Purpose | Content |
| --- | --- | --- | --- | --- | --- | --- |
| 1. Appropriate data collection methods were used. | 3. Staff involved were experienced, rigorous in their approach | 11. Generally the data provided was what I needed, although more participant and hospital details would have added to the value (eg age was in 5 year groups, which in young children is less than ideal). | 12. History | 13. I am aware of the data integrity checks that are done during and after data collection, and prior to data provision to researchers | 6. Data is collected for funding purposes | 7. Fields relevant for these reasons[funding] (such as timing of events are quite reliable). Other data, such as diagnoses, are unreliable as specific diagnoses (such as 'lobar pneumonia), but more reliable in larger aggregated groups (such as respiratory diseases) |
| 2. Audited annually | 10. For the VAED there are trained coders entering the information from patient notes | 14. Massive population based study, error would be across all data sets | 28. The Victorian State Trauma Registry was established and housed at Monash University | 18. Our ICU contributes data to this dataset & we implement similar quality checks to those of other ICUs. | 7. Data is provided for funding and governance reasons | 16. The survey items were not fit-for-purpose for our research, and the psychometric properties of the items had not been verified. |
| 3. Data were validated in several ways | 21. Small no. of data collectors, data collectors trained previous audits | 25. The data pertain to the population, it is not a sample. | 30. There are challenges with AIR data but it is pretty reliable. | 19. Fit for purpose requires an understanding of PHESS, and its limitations, to ensure fit as a good researcher | 9. Data were collected for routine administrative use | 17. However we have conducted validation and evaluated the cohort in the context of linked data and find it to be generally consistent, credible and thus fit for purpose. |
| 4. Coding and validation processes in place. | 28. Is well staffed by trained data collectors | 27. Population sampling for validity of outcomes. | 31.Errors are likely to be random and unlikely to be a source of bias. | 23.The data are easy to use once you understand what they mean | 26. The data was used for costing/billing for hospitals. There are problems, but it gave an idea of trends | 19. PHESS is used to access specific disease related data |
| 5. Data are collected in line with national coding rules and rigorous audit systems | 46. A highly dedicated team of health information managers and support staff, a midwife educator and epidemiologists oversaw the collection | N=4 | 33. Some variables are known to be less accurate. | 32. Was aware of regular quality assurance checks | 37. Yes as we were primarily using information from the dataset to recruit participants who had experience a major farm injury and participants who had contacted us did fit into this category. | 24. The data is relevant to our work and reflect real world |
| 6. From clinicians entering in field directly from a patient. | N=5 |  | 48. Reputable source | 35. We had a good understanding of what the case register could and couldn't tell us…. There are, of course, limitations with it, but we knew this going into the research and acknowledge these in outputs. | 43. Data were collected for administrative purposes. | 30. There are challenges with AIR data but it is pretty reliable. |
| 8.Data quality processes applied to collection |  |  | 22. The ANZICS APD has been used for numerous ICU studies | 36. We submit data from our unit to this registry. We are well aware of the quality processes that maintain the integrity of the data | N=6 | 39. Yes. The one variable I recall that could be improved relates to ethnicity - there were some inconsistencies - this is particularly important in a diverse state such as Vic. |
| 9. There is a level of quality assurance for administrative purposes |  |  | 35. It has been used extensively in prior research, leading to impactful outputs and outcomes | 47. Much of the data is qualitative. Its use requires a thorough understanding of how the data is collected. |  | N=7 |
| 13. Data integrity checks that are done during and after data collection, and prior to data provision to researchers |  |  | 49. Team publish data | N=8 |  |  |
| 15. Meticulous process to ensure data are obtained and entered by the Registry. |  |  | N=9 |  |  |  |
| 17. Not the processes conducted by the Department of Health or the VAED - I don't believe there are any. |  |  |  |  |  |  |
| 18. We implement similar quality checks to those of other ICUs. |  |  |  |  |  |  |
| 20. Process in place to regularly assess accuracy of data using computerised system |  |  |  |  |  |  |
| 22.While I was not able to personally validate it, I understand it has been validated |  |  |  |  |  |  |
| 23. I'm not sure what 'data quality processes' are meant here. |  |  |  |  |  |  |
| 27. The govt. organization responsible for data collection used state of art techniques in the field and laboratory and |  |  |  |  |  |  |
| 28. Robust routine quality assurance procedures |  |  |  |  |  |  |
| 29. There are a large number of checks on the data before it is released |  |  |  |  |  |  |
| 31. There is extensive validation at the time of data submission. Errors are likely to be random and unlikely to be a source of bias. |  |  |  |  |  |  |
| 32. Validation studies have been undertaken. |  |  |  |  |  |  |
| 33. Validation studies have quantified the accuracy. |  |  |  |  |  |  |
| 34. Vast majority of data points undergo validation |  |  |  |  |  |  |
| 36. Quality processes that maintain the integrity of the data |  |  |  |  |  |  |
| 40. There is a large number of checks undertaken on the data before it is released. |  |  |  |  |  |  |
| 41.Data custodians undertake cleaning and validation of the datasets, through various mechanisms (including cross referencing of multiple datasets and clinical review). |  |  |  |  |  |  |
| 42.Data quality relies on coding which has inherent issues, but in general provides a reliable high-level view of health care activity |  |  |  |  |  |  |
| 44.Efforts were made to ensure quality capture of information related to injuries so in terms of processes, data was captured that provided information as intended |  |  |  |  |  |  |
| 45. It is routinely collected data from all public hospital emergency departments. |  |  |  |  |  |  |
| 46. Coding and data entry undertaken with great rigor |  |  |  |  |  |  |
| N=29 |  |  |  |  |  |  |
|  |  |  |  |  |  |  |

*Green denotes positive comments; orange denotes neutral comments; red denotes negative comments*
