# Supplementary material for: The knowledge and reuse practices of researchers utilising government health information assets, Victoria, Australia, 2008–2020
Source: PLoS One. 2024 Feb 1;19(2):e0297396. doi: 10.1371/journal.pone.0297396 (PMC10833579; doi:10.1371/journal.pone.0297396)
Supplement: S1 Appendix — (DOCX) [file pone.0297396.s009.docx]

**S1 Appendix: Datasets for which a survey was completed (dataset-1 and dataset-2 combined)**

| **Dataset Acronym** | **Dataset Name#** | **Number** | ***Percent*** |
| --- | --- | --- | --- |
| ACIR/AIR | Australian Childhood Immunisation Register/Australian Immunisation Register | 2 | *2.7* |
| ANZICS APD | Australian and New Zealand Intensive Care Society Adult Patient Database | 9 | *12.2* |
| ANZPIC | Australian and New Zealand Paediatric Intensive Care Register | 1 | *1.4* |
| AuSCR | Australian Stroke Clinical Registry | 2 | *2.7* |
| BSV | BreastScreen Victoria | 1 | *1.4* |
| HACC MDS | Home and Community Care (HACC) Minimum Dataset | 1 | *1.4* |
| VAED | Victorian Admitted Episodes Dataset | 19 | *25.7* |
| VASM | Victorian Audit of Surgical Mortality | 1 | *1.4* |
| VCAR/VBDR | Victorian Congenital Anomalies Register/Victorian Birth Defects Register | 1 | *1.4* |
| VCCR | Victorian Admitted Episodes Dataset | 2 | *2.7* |
| VCOR | Victorian Cardiac Outcomes Registry | 1 | *1.4* |
| VCR | Victorian Cancer Register | 4 | *5.4* |
| VEMD | Victorian Emergency Minimum Dataset | 9 | *12.2* |
| VPCR | Victorian Psychiatric Register | 1 | *1.4* |
| VPDC | Victorian Perinatal Data Collection | 5 | *6.8* |
| VPHS | Victorian Population Health Survey | 5 | *6.8* |
| VSTR/VSTOR | Victorian State Trauma (Outcomes) Registry | 6 | *8.1* |
| VTP | Tuberculosis (TB) Undertakings Data Collection | 2 | *2.7* |
| Not stated |  | 2 | *2.7* |
| Total |  | 74 | *100* |

*#Within-scope datasets for which no survey responses were received included: Consultative Council on Obstetric and Paediatric Mortality and Morbidity (CCOPMM); Cardiac Surgery Registry (CSR); Elective Surgery Information System (ESIS); Life!; Victorian Consultative Council on Anaesthetic Mortality and Morbidity (VCAMM); Victorian Cost Data Collection (VCDC); Victorian Death Index (VDI); Victorian Eye Services Program Minimum Dataset (VES_MDS); Victorian Health Monitor (VHM); Victorian Radiotherapy Minimum Dataset (VRMDS).*
